# A Model for a Chikungunya Outbreak in a Rural Cambodian Setting: Implications for Disease Control in Uninfected Areas

**DOI:** 10.1371/journal.pntd.0003120

**Published:** 2014-09-11

**Authors:** Marguerite Robinson, Anne Conan, Veasna Duong, Sowath Ly, Chantha Ngan, Philippe Buchy, Arnaud Tarantola, Xavier Rodó

**Affiliations:** 1 Institut Català de Ciències del Clima, Barcelona, Spain; 2 Institut Pasteur du Cambodge, Phnom Penh, Cambodia; 3 National Dengue Control Program, Ministry of Health, Phnom Penh, Cambodia; 4 Institució Catalana de Recerca i Estudis Avançats (ICREA), Barcelona, Spain; United States Army Medical Research Institute of Infectious Diseases, United States of America

## Abstract

Following almost 30 years of relative silence, chikungunya fever reemerged in Kenya in 2004. It subsequently spread to the islands of the Indian Ocean, reaching Southeast Asia in 2006. The virus was first detected in Cambodia in 2011 and a large outbreak occurred in the village of Trapeang Roka Kampong Speu Province in March 2012, in which 44% of the villagers had a recent infection biologically confirmed. The epidemic curve was constructed from the number of biologically-confirmed CHIKV cases per day determined from the date of fever onset, which was self-reported during a data collection campaign conducted in the village after the outbreak. All individuals participating in the campaign had infections confirmed by laboratory analysis, allowing for the identification of asymptomatic cases and those with an unreported date of fever onset. We develop a stochastic model explicitly including such cases, all of whom do not appear on the epidemic curve. We estimate the basic reproduction number of the outbreak to be 6.46 (95% C.I. [6.24, 6.78]). We show that this estimate is particularly sensitive to changes in the biting rate and mosquito longevity. Our model also indicates that the infection was more widespread within the population on the reported epidemic start date. We show that the exclusion of asymptomatic cases and cases with undocumented onset dates can lead to an underestimation of the reproduction number which, in turn, could negatively impact control strategies implemented by public health authorities. We highlight the need for properly documenting newly emerging pathogens in immunologically naive populations and the importance of identifying the route of disease introduction.

## Author Summary

During the recent resurgence of chikungunya, the scale of imported cases into previously unaffected countries has caused great concern due to the presence of a competent vector (*Aedes albopictus*) in many of these regions. This study describes a mathematical model for a chikungunya outbreak in the rural Cambodian village of Trapeang Roka, where a chikungunya epidemic was recorded and documented in March 2012. The outbreak data is unique, in that all infections were confirmed by laboratory analysis, enabling the identification of asymptomatic individuals, in addition to individuals who failed to report details of their infection. A stochastic model, partitioning the infectious population into three distinct classes, is implemented using Gillespie's algorithm. We show that the incorporation of both biologically-confirmed symptomatic cases undocumented by date of fever onset and asymptomatic cases yields a higher estimate of the reproduction number. Our results highlight how reproduction numbers could be underestimated by limiting analysis to the epidemic curve. Carefully documenting cases and performing laboratory testing in cluster regions, such as the village considered here, could provide a more comprehensive insight into the true infection dynamics.

## Introduction

Chikungunya virus (CHIKV) belongs to the genus Alphavirus. It is a mosquito-borne pathogen transmitted by the Aedes mosquitoes. In humans, the virus causes an acute illness with symptoms including fever, headaches, rash and arthralgia [1]. The virus was first identified in Africa, during an outbreak in Tanzania in the 1950s [2]. In Africa the virus is maintained in a mainly sylvatic cycle, being spread among wild primates by forest dwelling mosquitoes [3], [4]. In contrast, in Asia the virus is spread between humans and the primary vector *Aedes aegypti* [5]. Chikungunya was first recorded in Asia in Thailand in 1958 [6]. Large epidemics were recorded throughout Asia in countries including Cambodia, Vietnam, Burma, Sri Lanka, India, Indonesia and the Philippines, before the virus virtually disappeared following the 1973 outbreak in India [5]. A large outbreak in Kenya in 2004 initiated a resurgence of the virus leading to widespread infection in the Indian Ocean islands of the Comoros, Seychelles, Mauritius and the French islands of Mayotte and La Réunion. The epidemiology of the virus changed, with the major vector on La Réunion identified as *Aedes albopictus* [7]. At the time, this was the largest documented outbreak, with over 266,000 cases estimated to have occurred [8]. Sizeable undocumented outbreaks were also observed in Asia and India during the 1960s but exact case numbers are unavailable [5]. Large outbreaks were detected in India in late 2005 [9], followed by outbreaks in Southeast Asia (Thailand, Singapore and Malaysia) in 2006 [10]. In 2011, a strain from the Asian lineage was reported in the Pacific island of New Caledonia, the first cases of chikungunya in this part of the world [11]. Countries in Europe, Asia and North America documented imported cases associated with travellers returning from India and the Indian Ocean islands [4], [12]–[14]. In 2007 the first chikungunya epidemic in a temperate country was recorded in the region of Emilia-Romagna in north-eastern Italy [15]. The virus was assumed to have been imported by a traveller from an infected region of India [16] and established itself in the local *Aedes albopictus* population, first detected in 1990 [17]. In September 2010, two autochthonous cases were documented in the southern French city of Fréjus where the *Aedes albopictus* vector is present [18]. There was no documented transmission beyond these two cases; whether the aggressive surveillance and control efforts implemented around those cases had a significant impact is unknown. In Cambodia, the virus was first detected in 1961, when the Asian genotype was circulating in the region [19]. From 2000, CHIKV serologies were performed at the Institut Pasteur in Cambodia on the samples collected by the dengue national surveillance and control program. The virus was first detected in Battambang province (Thai border) in 2011 and, since then, new cases have been reported in the country following a northwest-southeast direction [20].

Unlike dengue fever, which has been extensively modelled, chikungunya has only started to receive attention since its reemergence in 2005. Mathematical models have been developed to describe detailed mosquito dynamics and the host-vector interactions [21], [22]. A primary focus has been on determining the reproduction number, 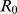, of an epidemic, which is defined as the number of secondary infections from an infected host in a completely susceptible population [23]. The standard approach has been to fit a dynamic model, with varying levels of detail describing the mosquito life cycle, to the epidemic curve. Such an approach has yielded various estimates for the La Réunion epidemic. Dumont and Chiroleu [24] obtained a value of 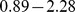 depending on the location on the island. They also considered the inclusion of increased mosquito mortality due to infection, yielding estimates of 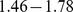 [25]. Considering seasonal fluctuations in the vector population, Bacaer [26] estimated a reproduction number of 3.4. More recently an estimate as high as 4.1 has been obtained [27]. A vastly different approach was adopted by Boëlle et al [28], who constructed the generation interval of chikungunya based on the gonotrophic cycle of the causative mosquito, obtaining a best estimate of 3.7, with a range of 2–11. A temperature-dependent host-vector model was fitted to the 2007 Italian outbreak by Poletti et al [29], estimating an 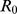 of 3.3 with a range of 1.8–6. Finally, the risk of chikungunya infection in an endemic dengue region was estimated to be 64% that of dengue with an 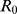 of 1.22 [30].

The scale of imported cases into previously unaffected countries (e.g. UK, France, Hong Kong, USA [4]) observed during the recent resurgence of chikungunya in the Indian Ocean has caused great concern due to the presence of a competent vector (*Aedes albopictus*) in many of these regions [31]. The threat of disease introduction is further compounded by the apparent ease at which the infection was established in the local Italian *albopictus* population during the 2007 outbreak. The urgent need to establish adequate monitoring and mosquito control programs in vulnerable countries is particularly highlighted by the recent outbreak in Singapore, in which 1059 cases were recorded in 2013 [32], despite a history of successful control measures to curb the transmission of this disease [33]. Recent work on the spatio-temporal spread of chikungunya through an immunologically naive population driven by asymptomatic individuals [34] underlines the risk of unknowingly importing the infection into new regions. With little information available in the early stages of an epidemic, estimates of the reproduction number are commonly used to inform public health decision makers and methods to obtain accurate estimates in newly infected regions are thus required to effectively assess the public health preparedness needs, the impact, and success of control measures.

The impact of asymptomatic cases and biologically-confirmed symptomatic cases with an undocumented date of onset is investigated in this paper. In March 2012, a local outbreak of chikungunya fever was reported in the rural village of Trapeang Roka in the Kampong Speu Province, Cambodia [35]. Chikungunya infection was confirmed by laboratory analysis allowing the identification of both asymptomatic and unreported cases. We formulate a stochastic model to describe the temporal dynamics of the outbreak and estimate the reproduction number by fitting the model to the recorded epidemic curve. The inclusion of biologically-confirmed cases undocumented by date of onset, which do not appear on the epidemic curve, allowed a more accurate estimate of the reproduction number to be obtained, in comparison to that obtained when such cases are excluded. This is the first attempt to apply such a stochastic model to a relatively isolated village typical of the Cambodian rural habitat, presenting the unique opportunity to consider the introduction of the virus into a comparatively closed and immunologically naive population.

## Methods

### Ethics Statement

The data collection protocol, implemented on March 26 2012, in Trapeang Roka village was validated by the Cambodian Ministry of Health. Informed consent was obtained in writing from all adults in the Khmer language and parents were asked to sign for their children.

### Description of the Cambodian Outbreak

An outbreak investigation was conducted on the 26th of March, 2012, after reports of illness, consisting of fever and rash, among residents of Trapeang Roka village were confirmed by blood samples to be CHIKV infection [35]. The population of the village was estimated to be 695 individuals, living in 134 houses. The investigation protocol was validated by the Cambodian Ministry of Health. As part of the investigation, 98 houses were visited and 425 people were interviewed. Adults were asked for their consent and the consent for their children. All the people in the visited houses were asked to complete a standardized questionnaire in the Khmer language. Questions were about demographic data (such as age and sex), socio-economical data (such as occupation and level of education) and clinical data during the previous 6 weeks, corresponding to the time period since the rains occurred. Clinical data included observed symptoms (skin rash, joint pain, temperature) and the date of symptom onset. Blood was collected on blotting paper for each individual. A venous blood sample was performed on febrile individuals. Samples were sent to the Institut Pasteur in Cambodia. Serology by IgM-Capture Enzyme-Linked Immunosorbent Assay (MAC-ELISA) to detect IgM against CHIKV was performed on dry blood spots [36]. No follow up blood test was performed on sero-negative people to observe seroconversion. The blood of febrile individuals was tested by RT-PCR for CHIKV [37]. The chikungunya variant E1-226V strain was identified [20]. A positive case was defined as a person who had at least one sample which tested positive for CHIKV (IgM serology and/or RT-PCR). Serologic testing was also performed for flavivirus antibodies. Anti-dengue virus or anti-Japanese encephalitis virus antibodies were detected in 20 CHIKV seropositive people. The epidemic curve was built with the number of biologically-confirmed CHIKV cases per day determined from the date of fever onset, which was self-reported in the questionnaire.

**Figure 1:**
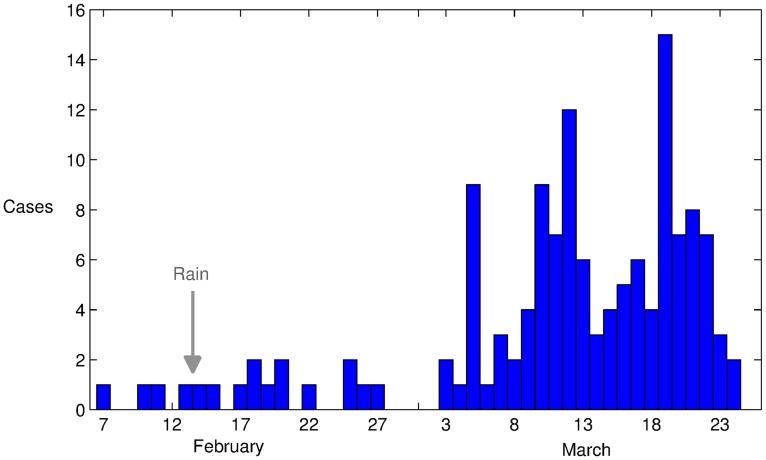
Epidemic curve showing confirmed chikungunya per day by date of reported onset in the village of Trapeang Roka, Cambodia. The grey arrow indicates the start of a two-day rain spell.

The data spanned a period of 48 days between February 7 and March 25 inclusive, the the final laboratory confirmed cases of chikungunya, with a clinical onset, detected on March 24, Figure 1. It has been documented previously that chikungunya outbreaks often follow large rainfall episodes [38]–[40], which result in a surge in the local mosquito population [41]–[44]. The dominant mosquito population identified in the region during an entomologic assessment performed on March 29-30 was *Ae. aegypti* [35]. The rains arrived on February 14 and persisted for 2 days. Following the rains and the associated increase in water availability for oviposition, the time delay between the rains and the epidemic gaining momentum (approximately 16–18 days) is consistent with the duration of the larval/pupal stages (12.5 days) [45] and the minimum egg incubation period (3 days) [46].

**Figure 2:**
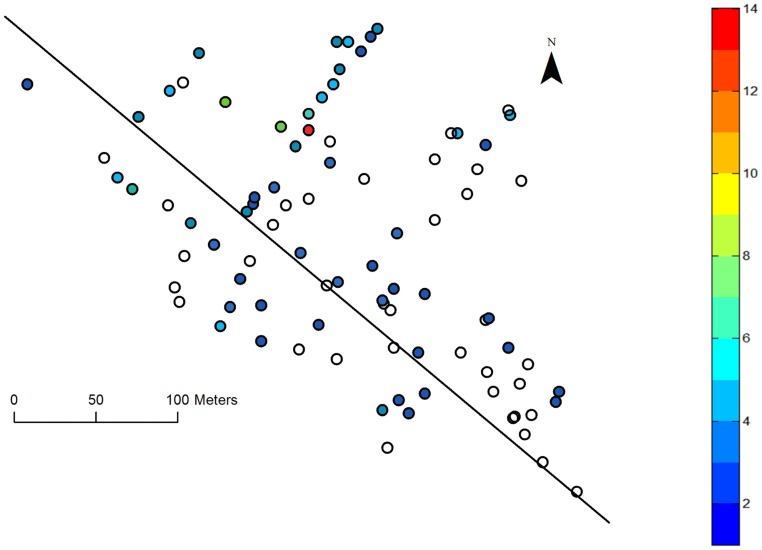
A map of Trapeang Roka village, showing all houses for which gps co-ordinates were collected. The map shows the distribution of biologically-confirmed symptomatic cases, documented by date of fever onset. Each circle denotes the location of a house within the village. Unfilled circles indicated houses that escaped infection. The colour bar indicates the number of symptomatic cases with a documented date of symptom onset in each house. The black diagonal line indicates the main road running through the village, about which the houses are clustered.

**Figure 3:**
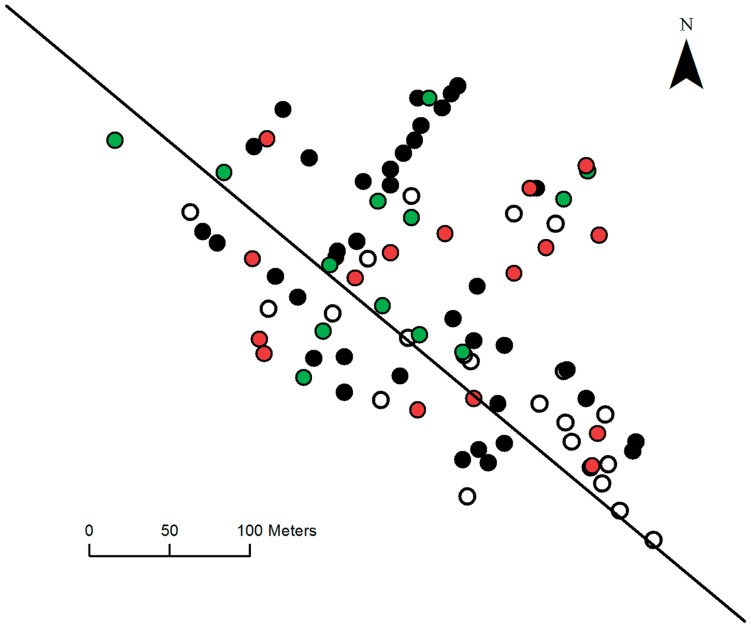
A map of Trapeang Roka village, showing all houses for which gps co-ordinates were collected. The map shows houses with no confirmed infection (unfilled circle), houses with only infections documented by date of onset (black circle), houses with only infections undocumented by date of onset (red circles) and houses which have both cases with documented and undocumented infection onset dates (green circle). The black diagonal line indicates the main road running through the village, about which the houses are clustered.

Data collection took place on March 26, and it is likely that there were some cases after this date. The 98 houses visited were randomly located throughout the village, with longitude and latitude recorded for all but 10 houses (Figure 2). Of the 425 individuals interviewed, 190 laboratory confirmed cases of chikungunya were detected, 5% of which were asymptomatic. The date of symptom onset was recorded for 138 of the confirmed cases and 52 individuals were either asymptomatic (10 individuals) or could not recall the date of symptom onset. The outbreak consisted of an initial period, between epidemic days 1 and 25, during which sporadic cases occurred but with no consistent growth pattern. It is noted that the outbreak itself struck houses at random throughout the village and was not spatially restricted to a particular region (Figure 2). The high incidence observed in four houses located in the north of the village can be attributed to their above average household sizes in the range 6–13 individuals per house (village average 4.3). A single infectious mosquito in such a house has many hosts available for feeding and would thus be capable of transmitting the infection to a greater number of individuals. As the data collection occurred 7 weeks after the index case, it is possible that the 42 individuals who failed to recall their specific infection details, despite testing positive for infection, may have been infected in the earlier period of the epidemic. Another possibility is that these individuals suffered minor illness and, as such, could not recall specific details. The location of these individuals, undocumented by date of onset, does not form an isolated cluster within the village and they are distributed randomly among the houses surveyed, Figure 3.

**Figure 4:**
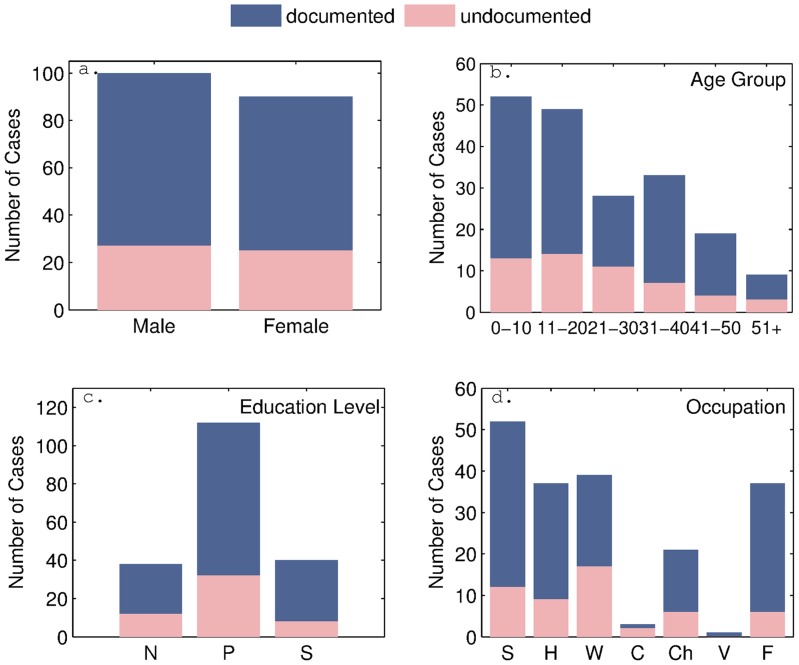
Bar charts showing the distribution of cases with documented and undocumented (including asymptomatic) dates of symptom onset within the population. (a) gender of cases, (b) age group of cases in years, (c) education level of cases: No schooling (N), Primary school (P), Secondary school (S), (d) occupation of cases: Student (S), Stay at home (H), Factory worker (W), Construction worker (C), Child (Ch), Vendor (V), Farmer (F).

Finally, we considered any bias that may have caused people to not report dates of symptom onset, Figure 4. We found that gender did not play a role, with both men and women equally likely to report infections. Age also did not appear to be a significant factor, with only slightly lower reporting rates amoung 31–50 year old groups. Surprisingly, individuals with a secondary level education were less likely (22%) to report their infection in comparison to those with no schooling (32%) or a primary level education (29%). Students and homemakers were more likely to report symptoms, a fact that could perhaps be attributed to their increased likelihood of being present during the data collection campaign. In fact, this could possibly also explain the increased reporting rates amoung middle aged people with secondary level education. This indicates that the time of day the data collection takes place may play a factor and is likely to omit people working outside the home or village.

### Model Formulation

The disease dynamics are modelled by considering both host and vector populations explicitly. The human population is divided into susceptible (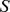), exposed (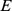), infectious (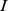) and recovered (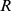) individuals. The outbreak was short (7 weeks) relative to the human lifespan and the total human population is taken to be constant, 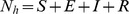, and it is assumed that the exposed population is not infectious. The data did not record the date of symptom onset for 52 laboratory confirmed cases. Of these 52 individuals, 10 were asymptomatic and the remainder could not recall the exact date of onset, possibly due to the seven week lapse between the epidemic outbreak and the data collection campaign. Nonetheless, these cases will be incorporated into the model as these individuals are also capable of transmitting the infection.

Firstly, it is assumed that, following the latent phase, both asymptomatic and symptomatic cases become infectious at the same time. It is also assumed that the latent and incubation periods coincide, so that exposed individuals are not infectious to biting mosquitoes. This may not be strictly true for chikungunya, but a definitive consensus on the relative lengths of these infection states has not been reached to date [47]. Unlike directly transmitted diseases, such as influenza or measles, the absence of symptoms does not necessarily decrease the likelihood of transmitting the infection. There is a possibility that a lower viral load in asymptomatic individuals may decrease their ability to transmit the virus to susceptible mosquitoes, however, these is no evidence to confirm this theory and, in fact, the difference in viral loads observed between symptomatic and asymptomatic individuals have been shown to not be statistically significant [48]. In addition, there is documented evidence of virus transmission from symptomatic seropositive primates to seronegative animals via experimental mosquito bites [49] at viremic levels detected in asymptomatic humans. Furthermore, viremia levels in asymptomatic cases are sufficiently high as to cause widespread concern for possible contamination of donated blood supplies [16], [50]. Other factors could also lead to differences in the transmission potential of individuals. For example, self-imposed quarantine of clinical cases could reduce the mobility of symptomatic people and reduce transmission to other houses in the village. Therefore, we assume that both infectious states transmit the virus at the same rate but will consider a reduced transmission rate for asymptomatic individuals in a sensitivity analysis. However, the asymptomatic cases, lacking overt clinical presentations, avoid detection and act as silent spreaders within the population. The symptomatic cases with undocumented dates of onset, while overtly presenting clinical symptoms, are also not visible on the epidemic curve but are equally likely to infect a susceptible mosquito. To this end, the infectious compartment is separated into three sub-compartments, individuals who are asymptomatic 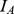, those who are symptomatic but undocumented by date of onset 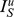 and those who are symptomatic and documented by date of onset 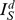. The total number of infectious individuals can thus be written as 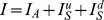. Finally, it is assumed that the recovery rates for each of the infectious states are identical. All of the above assumptions are commonly used in dynamic models for chikungunya fever [22], [27].

The stochastic nature of the infection process becomes important in small populations or when the number of infectious individuals is relatively small [51]. In a village of less than 1000 individuals a stochastic modelling framework is appropriate and is adopted herein. The deterministic equations, from which the stochastic model can be easily derived, for the human population are
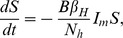


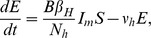


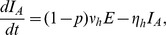


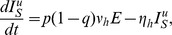


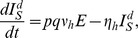


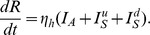



The susceptibility of a human to infection following a bite from an infectious mosquito is denoted by 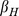. 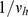 and 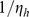 are the human incubation and infectious periods respectively, and 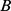 is the average daily biting rate of the mosquito. The proportion of infected individuals who develop symptoms is denoted by 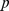 and 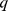 is the proportion of symptomatic individuals who are documented by date of onset.

**Figure 5:**
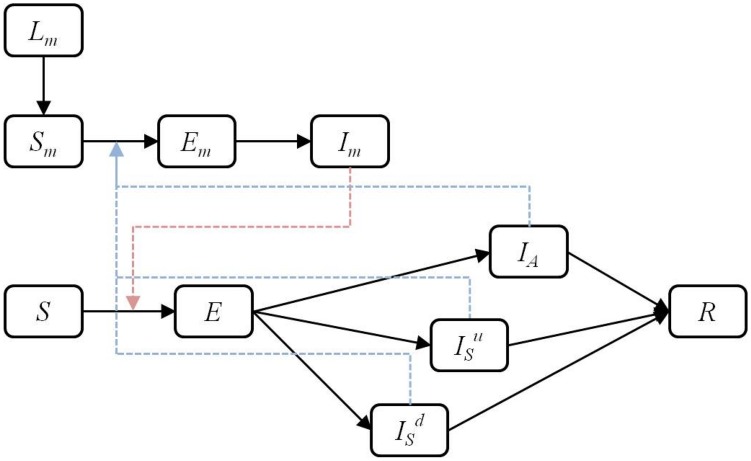
Schematic of the disease transmission pathway. Black arrows indicate transitions between disease states. A susceptible mosquito (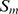) can be infected by a symptomatic human documented by date of onset (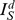), a symptomatic human undocumented by date of onset (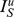) or an asymptomatic (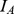) human (dashed blue arrow). A susceptible human (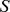) can be infected by an infected mosquito (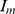) (dashed red arrow).

Following Dumont et al [24], [25], the adult female mosquito population is divided into susceptible (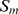), exposed (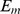) and infectious (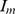) mosquitoes. A larval compartment (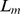) is included to describe the dynamics of the immature mosquito populations (Figure 5). The mosquito dynamics are described by
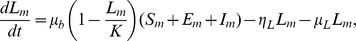


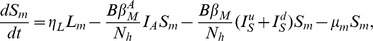


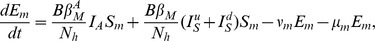


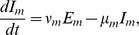



where 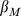 is the susceptibility of the mosquito to infection after biting a symptomatic infectious human and, similarly, 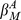 is the susceptibility of the mosquito to infection after biting an asymptomatic human. The transmission rates will be treated as equal, 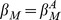, but a lower transmission from asymptomatic individuals will be considered later in a sensitivity analysis. 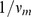 is the mosquito latent period and 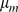 is the adult mosquito natural death rate. It is assumed that mosquitoes do not recover from infection. 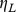 is the maturation rate of the immature mosquito population. 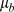 is the average number of female eggs laid per day per adult female mosquito and 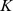 is the carrying capacity, the maximum population of immature mosquitoes that can be sustained by the available resources. 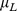 is the natural larval mortality rate.

The basic reproduction number can be easily calculated from the next generation matrix [24] to obtain
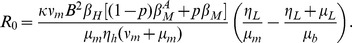



For the stochastic version of the model, all the continuous variables become discrete numbers and each compartmental transition becomes a distinct event with an associated rate. There are 16 distinct events in the stochastic model which are listed in Table 1.

**Table 1:** Events and rates in the stochastic model.

Event	Transition	Rate
mosquito-to-human infection	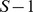 , 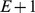	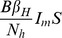
onset of asymptomatic infection	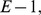 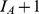	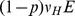
onset of undocumented symptomatic infection	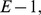 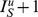	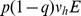
onset of documented symptomatic infection	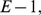 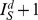	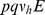
recovery from asymptomatic infection	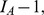 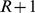	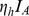
recovery from undocumented symptomatic infection	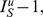 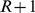	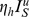
recovery from documented symptomatic infection	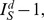 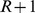	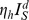
mosquito egg deposition	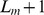	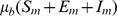
egg death due to resource limitations	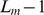	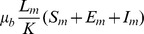
mosquito maturation	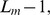 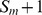	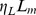
larval death	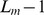	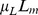
human-to-mosquito infection	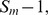 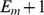	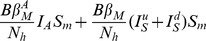
onset of infectiousness	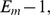 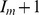	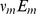
susceptible mosquito death	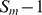	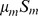
exposed mosquito death	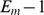	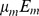
infected mosquito death	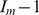	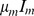

### Model Parameters

The human population is set to the sample size, 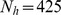. The latent period in the human population is the time from a mosquito bite to the onset of infectiousness, which for the clinical cases is assumed to coincide with the onset of symptoms. The incubation period for chikungunya can range from 2 days to 12 days, with a mean of approximately 3–7 days [1], [8], [28], [47], [52]. The value 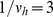 days is used in this work, which is comparable with values used in other modelling studies [24], [25], [29]. The duration of infection for chikungunya is typically in the range 1–7 days [5], [53] but symptoms can persist for several weeks [8]. The data collection campaign conducted in the village recorded both the date of fever onset and the date the fever resolved. The mean febrile period was calculated as 4.28 days with a standard deviation of 2.5. The duration of the infectious period was thus taken as 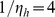 days. The proportions 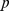 and 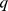 are estimated from the data to be 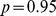 (yielding 10 asymptomatic individuals in an infectious population of 190) and 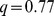 (yielding 138 cases which are documented by date of onset out of a total of 180 symptomatic cases.)

Biting rates for *Ae. aegypti* have been measured in laboratory settings with average values of 0.7 bites per day [54], [55]. However, they have been shown to be opportunistic feeders with biting frequency increasing with host availability [56]. Taking a conservative estimate, we limited host availability to 12 hours per day and the resulting biting rate is approximately 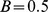 [54]. Laboratory experiments, in which *Ae. aegypti* were infected orally with chikungunya variant E1-226V, detected the virus in the salivary glands 2 days after infection [57], indicating a mean extrinsic incubation period of 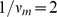 days. The susceptibility of mosquitoes to infection following a blood meal has been extensively studied. *Ae. aegypti* mosquitoes challenged with a strain of the virus from the La Réunion epidemic displayed infection rates of 88.5% to 90.7% [57]. *Ae. aegypti* from the French West Indies and French Guiana, infected by blood with a titre of 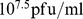 showed infection rates from 88.9% to 100%, but as low as 37.6–62% when infected with a titre of 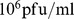 [58]. Girod et al found that infection rates were found to depend heavily on the housing density of the region where the mosquito was captured, with dense housing yielding an infection rate of 56.8% and diffuse housing a rate of 38.2%. However, these results were for mosquitoes collected from chikungunya-free regions, where local transmission has not been documented to date. Mosquitoes from Cameroon and Vietnam, exhibited infection rates of 37.1–84.8% and 66.5–99.6% respectively, when challenged with several viral strains [59]. In particular, when challenged with the East/Central/South African strain (06.117), which was identified in this outbreak [35], the Cameroon and Vietnamese mosquitoes displayed infection rates of 64.8% and 78.3% respectively. Mourya et al [45] found that, at 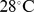 and relative humidity 70–80%, the rate of infection was 61.82%. Such values are comparable with conditions in the relevant region of Cambodia during March, with a long-term average from 1981 to 2013 yielding a temperature of 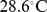 (range 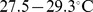) and relative humidity in the range 75–83%. A conservative value of 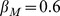 is taken in this work. No studies have been conducted on human susceptibility to infection following a bite from an infected mosquito and the value of 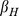 will be estimated from the epidemic curve.

All other parameters are intrinsically linked with the mosquito life cycle. The lifespan of the female *Ae. aegypti* mosquito under various temperatures has been measured in a laboratory setting [45]. Observations indicate that the adult female has a mean survival duration of 43.7 days at 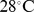 but this is considerably reduced to 18.17 days at temperatures up to 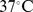. However, the controlled environment of such laboratory studies will undoubtedly overestimate the life span. Mark-release-recapture studies performed on wild *Ae. aegypti* populations in Rio De Janeiro during the wet season with temperatures in the range 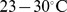 estimate an average life expectancy (ALE) of 3–16 days [60], with this being limited to 1.9–5 days in high income neighbourhoods [61]. Similar studies in Kenya calculate a mean survival of 9 to 10.7 days [62], [63]. Studies in Northern Australia, performed in the temperature range 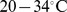, found the probability of daily survival (PDS) to be 0.86–0.91 [64], which yields an ALE in the range 6.6–10.6 days (using the relation 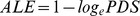 [65]). A value of 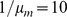 days is used in this analysis. A study in Malaysia found that *Ae. aegypti* produced an average of 86 eggs per oviposition [66]. The mean gonotrophic cycle length was found to be 3 days, which yields an average of approximately 3.3 cycles in a 10 day lifespan and a lifetime total of 286 eggs. Thus, the breeding rate per female mosquito is approximately 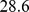 eggs per day. Assuming approximately half of all eggs laid result in the emergence of a female mosquito [45] then the breeding rate is 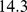 eggs per day. Finally, for temperatures in the range 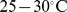, 29.6% of eggs fail to hatch [67] yielding 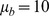 per day. Laboratory measurements by Mourya et al [45] for the duration of larval stages indicated a length of 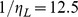 days to the emergence of the adult mosquito. Furthermore, they found that larvae and pupae experience 2% and 6.63% daily mortality rates respectively. In the wild, larval mortality will be dependent on many factors such as the destruction of breeding sites, moisture levels, temperature and interspecific competition [68]. As such, we take the upper limit of the mortality range and set 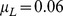.

Finally, following Dumont et al [24], the carrying capacity of the immature mosquito population 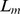 is taken to be a multiple of the human population 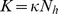, where 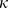 is the total number of immature mosquitoes per human. Surveys performed in Cambodia during the months August to October in areas at high-risk for dengue outbreaks found that the number of pupae in households was highly correlated with the adult mosquito population [69]. The mean pupae density was 16.4 per house, with a distribution ranging from 5.2/house in the rural area of Takeo province and up to 56.9/house in a rural area of Battambang, both comparable to the study site. In rural areas the pupae per person index is 3.6 and this was found to be independent of the human population density and the distribution of water containers [69]. We assume that the number of larva per person can be inferred from this; taking into account a 2% larval mortality rate and a 50% male-female ratio [45], we obtain 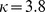 larva per person at the start of the outbreak. All parameters used in the simulations are summarised in Table 2.

**Table 2:** Parameters and values used in numerical simulations.

Parameter	Description	Value	Reference
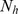	Human population	425	-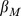	Mosquito susceptibility to infection from symptomatic	0.6	[45], [59]
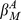	Mosquito susceptibility to infection from asymptomatic	0.6	-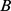	Average daily biting rate	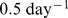	[54]
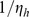	Mean viremic period	4 days	-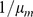	Average lifespan of adult mosquitoes	10 days	[60], [62]–[64]
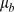	breeding rate of females mosquitoes	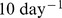	[66]
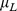	Natural mortality of immature mosquitoes	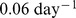	[45]
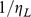	Duration of larval stage	12.5 days	[45]
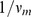	Extrinsic incubation period	2 days	[57]
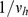	Intrinsic incubation period	3 days	[1], [8], [28], [47], [52]
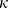	Number of immature mosquitoes per human	3.8	[69]
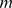	Number of female mosquitoes per human	1.2	[70], [71]
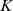	Maximal larval capacity	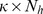	[24]

### Model Implementation

The epidemic curve, Figure 1, indicates the presence of a single documented symptomatic case on epidemic day 1 (February 7) yielding an initial condition with 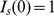. Following Dumont et al [24], [25], the mosquito abundance is taken to be dependent on the total human population present, such that initial mosquito populations are taken as
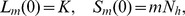
where 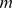 is the number of adult female mosquitoes per human. For a typical village in South East Asia, consisting of wooden houses, measurements indicate a population of 14.2 mosquitoes per house [70]. The village in the present study has the same construction characteristics and consists of a total of 134 houses, yielding an average of 2.7 mosquitoes per person. Furthermore, investigations found that, in December, the percentage of female mosquitoes was 42% [71]. Thus, the number of female mosquitoes per human in the village is approximately 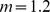. It is assumed that there is no pre-existing immunity in the village population as no other chikungunya outbreaks had been recorded in the village or in Cambodia for 50 years, as attested by the attack rate which remained at 50% until the age of 50, after which it dropped dramatically [35].

The stochastic process detailed in Table 1 is implemented using Gillespie's tau-leap algorithm, such that in a given time interval 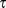 the event rates are calculated and the number of transitions in the interval 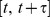 are evaluated. For each realisation of the model, the number of new symptomatic cases documented by date of onset on a given day 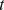 is determined from
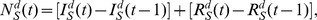
which, in the deterministic framework, corresponds to the integral
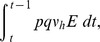
and where 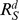 denotes the individuals who have recovered from a symptomatic infection and who were documented by date of onset. The objective function 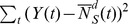, where 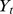 is the number of newly documented cases on day 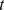 given by the data and 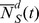 is the mean of 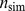 realisations of the model, was minimized using the *patternsearch* routine included in the Matlab Global Optimization Toolbox [72], [73]. The number of realisations 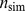 was chosen to ensure convergence. Each stochastic realisation is permitted to run for a maximum of 1 year or until the population is infection free, such that the following condition is satisfied:
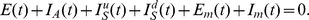



For the realisations where the duration of the simulated epidemic exceeds that of the observed epidemic it is assumed that no cases occurred after March 24 such that the data is padded with zeros to enable calculation of the objective function. 95% confidence intervals were calculated by performing a latin hypercube sampling of the parameter space using 1000 samples and minimizing the objective function using the deterministic version of the model.

## Results

On inspection of the epidemic curve, Figure 1, it can be seen that additional symptomatic cases of chikungunya fever were recorded from as little as 2 days after the initial index case on February 7. As such, given the extrinsic (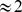 days) and intrinsic (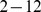 days) incubation periods, it is not possible that the recorded index case could have caused secondary infections so rapidly. Therefore, the data itself indicates that this was not the true index case. It is possible that either this individual incorrectly recorded the date of symptom onset (collected 7 weeks after the event) or the infection was already present in the population before February 7. Infection with another pathogen is also possible, however, the index case tested negative for other flaviviruses. We adopt the hypothesis that the infection was already present in the population on February 7 and to reflect this the initial conditions which are considered herein are
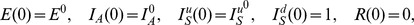


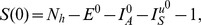


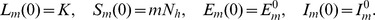



Fitting the stochastic model to the data yields estimates of the initial infected human population of 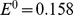 (95% C.I. [0.08–1.08]), 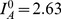 (95% C.I. [0.27–5.24]) and 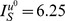 (95% C.I. [2.68–9.46]). The infected mosquito populations were estimated as 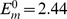 (95% C.I. [0.29–5.28]) and 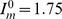 (95% C.I. [0.25–4.26]). The results corroborate our earlier observation that infection was certainly more widely spread within the population on the recorded outbreak day (February 7) and indicate that the infection was present in at least 8 other members of the population. These infectious individuals, being asymptomatic or undocumented by date of symptom onset, would have escaped detection and could account for the symptomatic cases observed on epidemic days 4 and 5. Many of the villagers worked outside of the village raising the possibility that these early secondary cases were in fact imported from other local infected regions. However, it is unlikely that such a large number of cases were simultaneously imported into an uninfected village. Furthermore, our model estimates that approximately 3 infected mosquitoes were also circulating within the village on February 7. Due to the limited flight range of the causative mosquito (maximum of approximately 500 m [74]) they could have been infected outside and transported by road into the village and thus seeded the epidemic. Alternatively, they could have been infected within the village. In the latter scenario, the duration of the extrinsic incubation period indicates that the infectious mosquitoes were infected at least 2 days before February 7. This indicates that a visitor to the village or a local who was either asymptomatic or undocumented by date of symptom onset may have imported the infection, which was then established in the local mosquito population, leading to the recorded index case.

**Figure 6:**
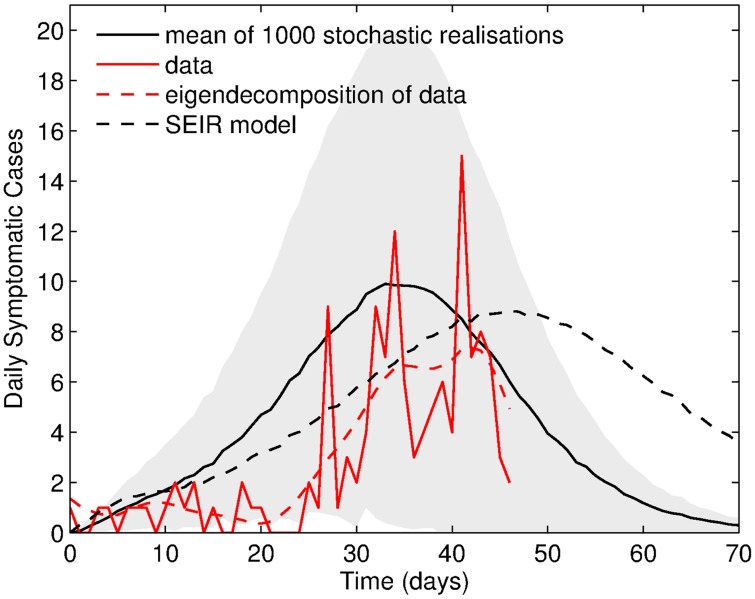
The mean of 1000 stochastic realisations for the number of daily symptomatic cases documented by date of onset (solid black line) plotted with the epidemic curve (solid red line). Also shown is the mean of 1000 realisations of the SEIR model (dashed black line) and an eigendecomposition of the epidemic curve (dashed red line). The grey shaded area shows the 95% confidence interval. Day 0 corresponds to the start of the epidemic on February 7.

**Figure 7:**
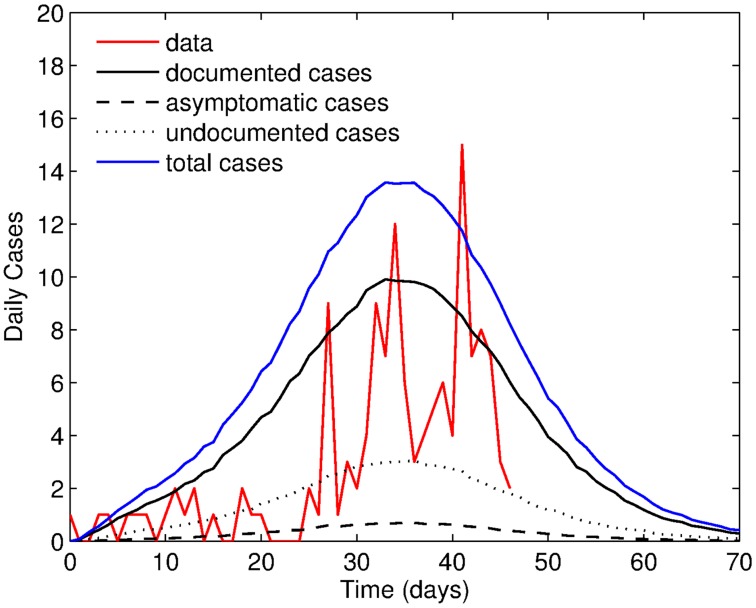
The mean of 1000 stochastic realisations for number of symptomatic cases documented by date of onset (solid black line), symptomatic cases undocumented by date of onset (dotted black line), asymptomatic cases (dashed black line) and the total number of infectious cases (solid blue line) plotted with the epidemic curve (solid red line). Day 0 corresponds to the start of the epidemic on February 7.

Finally, the data fitting procedure estimated the human susceptibility to infection as 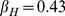 (95% C.I. [0.41–0.45]), which yields an estimate for 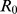 in Trapeang Roka village in February-March 2012 of 6.46 (95% C.I. [6.24, 6.78]). The daily cases are plotted, along with the epidemic curve, in Figure 6. An initial slow rise of the simulated epidemic can be observed which mirrors the true epidemic progression. However, the simulated epidemic does not wane near epidemic day 20 on February 27 (see epidemic curve in Figure 1). The timing of the epidemic peak shows a good comparison with the data and the simulated epidemic starts to decline in line with the epidemic curve. In addition, an eigendecomposition was applied to the chikungunya symptomatic cases time series to ease comparison with the model estimate. This partition of variance by strength of components is an effective method to separate signal from noise particularly in short time series such as the one under investigation. The covariance matrix equivalent of processing a forward prediction data matrix is generated with an eigendecomposition order of 20. Above 60% of the overall variability was accounted for by this reconstructed component to which the average model estimate can be compared. The eigendecomposition shows two distinct epidemic periods, the initial low level outbreak followed by the main epidemic event. Our model displays the initial slow growth, peaks several days earlier but shows a similar declining trend. The simulated solutions for the three infectious states (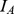, 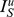 and 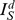), together with the total number of infectious individuals are shown in Figure 7. The curves show that an underestimate of approximately 5 cases at the peak is made by neglecting asymptomatic cases and those undocumented by date of symptom onset.

In place of daily cases, the cumulative cases could have been used for the estimation procedure. Some authors believe that this presents an advantage as it can smooth out demographic noise and avoid sociological factors that lead to noisy data, such as inaccurate reporting [75], [76]. We ran the fitting procedure using the cumulative cases which yielded an estimate of 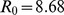 (95% C.I. [8.39–8.88]). This larger value appears to result from the rapid increase in cases in early March that follows a relative slow growth phase throughout February and imparts a steep slope on the cumulative case numbers. The parameter estimates obtained in this case were 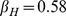 (95% C.I. [0.56–0.59]), 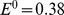 (95% C.I. [0.06–0.82]), 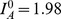 (95% C.I. [0.27–5.44]), 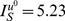 (95% C.I. [2.56–9.37]), 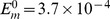 (95% C.I. [0–2.1]) and 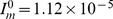 (95% C.I. [0–2.09]). Apart from the mosquito populations, these estimates are comparable with the results obtained using the daily case numbers. The negligible mosquito populations could be attributed to the lack of significant disease spread during February. This imparts a very low number of cumulative cases initially, which the model achieves by having negligible infected mosquito populations, and hence low virus transmission.

To investigate the impact of including compartments for the asymptomatic individuals and those undocumented by date of onset in the model structure we reconsider the estimation procedure using a simplified SEIR model. This essentially corresponds to the special case where 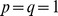, so that only symptomatic cases documented by date of onset are considered. A simple integration of the model yields 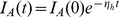 and 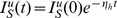, and we take 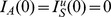 so that the populations of these two compartments are identically zero. This model yields a much lower 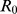 value of 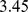 (95% C.I. [3.37–3.88]). However, the human and mosquito population estimates are 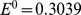 (95% C.I. [0.05–2.22]), 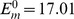 (95% C.I. [14.07–19.85]) and 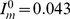 (95% C.I. [0.02–1.94]). The simulated epidemic under these conditions is presented in Figure 6. The curve shows a slower rise in case numbers which appears to fit the data better, however, the simulated epidemic continues long after the real epidemic has finished. The estimated population of initial exposed mosquitoes is particularly unrealistic given the lack of infection in the human population. This high value is required by the model to ignite the epidemic due to the absence of individuals who are either asymptomatic or undocumented by date of onset in the population.

**Figure 8:**
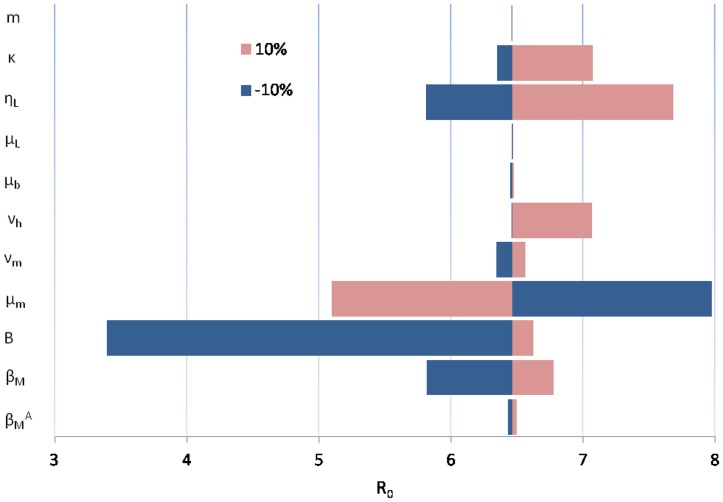
Tornado diagram of univariate sensitivity analysis. The diagram shows the degree to which a 10% variability in the parameters affects the value of 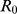. Each bar is a representation of how uncertainty in that particular parameter affects the estimate of the reproduction number. The baseline scenario is fixed with 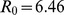.

A sensitivity analysis was conducted to identify the most influential parameters. The results of a univariate analysis are displayed in Figure 8. The model parameter that had the greatest influence on 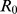 is the biting rate 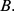 A 10% decrease in B yields an estimate as low as 3.4. The model is less affected by increases in 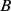, with a 10% increase yielding 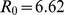. Significantly larger estimates of 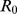 are obtained if the mosquito lifespan is extended (i.e. lower 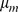). Permitting adults mosquitoes to survive longer enables them to continue spreading infection for longer periods of time yielding 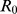 estimates as high as 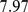 for a 10% decrease in 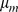. Other model parameters that show a substantial impact on the estimate of 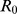 are the duration of the larval stage, 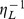, the mosquito susceptibility to infection, 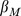, and the number of immature mosquitoes per human, 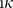. Notably, the solution is not sensitive to variations in the transmission potential of asymptomatic individuals, 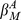. Undoubtedly, this is due to the small proportion of asymptomatic infection in the population, only 5%. This result justifies the earlier model simplification that asymptomatic individuals transmit as efficiently as those exhibiting symptoms. Other recorded chikungunya outbreaks have estimated larger asymptomatic prevalence, in the range 16.7–27.7% [77]–[79], and in such cases the impact of this parameter may be significantly more pronounced. Other parameters that exert little influence on 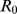 are the number of adult mosquitoes per human, *m*, the natural mortality of immature mosquitoes, 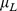 and the mosquito breeding rate 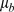.

## Discussion

In this paper an estimation procedure for the basic reproduction number of a chikungunya outbreak in a rural Cambodian village has been presented. This isolated outbreak is particularly important because it presents the opportunity to analyse the idealised situation of the importation of a new pathogen into an isolated and immunological naive population. The outbreak data is unique amongst other chikungunya outbreaks, in that all infections were confirmed by laboratory analysis, enabling the identification of asymptomatic individuals, in addition to individuals who failed to report details of their infection. This provided us with the opportunity to develop a novel mathematical model in which both symptomatic cases undocumented by date of disease onset and asymptomatic cases were incorporated. We found that the infection was already established in the village population on the reported epidemic start date, in the form of cases with an undocumented date of onset and asymptomatic cases. The presence of such individuals can lead to inaccuracies in the calculation of the basic reproduction number, which we showed using a simplified model excluding these cases. A rapid implementation of data collection campaigns, including laboratory analysis of all exposed individuals, in newly infected regions could possibly reduce such inaccuracies by allowing identification of the route of disease importation. Such inaccuracies in data collection could have a large impact on the implementation of control measures by public health authorities, who commonly rely on the reproduction number to inform decisions in the early stages of an epidemic.

The epidemic displayed a bimodal character, with a minor outbreak in February followed by the main outbreak in March. A similar structure was observed in La Réunion Island, which was due to a viral mutation and spanned months rather than weeks. In this case, we believe the arrival of the rains may have impacted the epidemic timing. The structure of our model is unable to reproduce this bimodel behaviour without an external forcing source. The inclusion of rainfall data as a mechanism for mosquito reproduction can produce such behaviour and this has been shown elsewhere using an agent-based model (ABM) [34]. Further work on understanding the dynamics of this outbreak could include a spatial analysis using the ABM framework to incorporate the topography of the village and local rainfall data to drive mosquito reproduction.

The model results highlight the importance of accurate epidemiological data collection to identify the route of disease importation and the ability of poor human recall to impact the epidemic curve by excluding cases with an undocumented date of onset. The consequences of excluding these cases was demonstrated by considering a simplified SEIR model which yielded biologically unrealistic mosquito population estimates and produced a simulated epidemic that continued for long after the real epidemic had finished. Furthermore, many individuals interviewed recalled a date of symptom onset but infection was not confirmed by IgM. This indicates that biological confirmation is crucial to avoid errors introduced due to the presence of other diseases (e.g. dengue, Japanese encephalitis), the impact of memory bias and the unintentional effects of people aiming to please the interviewers by confirming a non-existent infection. The results demonstrate that the identified index case was not alone in the population at the epidemic onset and the infection was already present in both the human and mosquito populations. The infected humans could have imported the infection from nearby regions, however, it is more probable that the infection was imported by an infected visitor or a resident who was either asymptomatic or undocumented by date of symptom onset, who would not have been recorded on the epidemic curve but nonetheless seeded the epidemic within the village's mosquito population.

The estimate obtained for 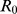 is larger then, but within the range, of values estimated for other chikungunya outbreaks in La Réunion [28] and Italy [29]. We believe this is largely due to the specific characteristics of the village, where large household sizes prevail in a small relatively isolated location. This resulted in an outbreak that was both quick and severe, with the infection sweeping rapidly through the village, infecting 44% of inhabitants within 7 weeks. It has been suggested that the basic reproduction number increases with mean household size [80]. This effect can be contributed to a larger quantity of water being stored to accommodate a greater number of people for daily tasks such as eating, washing and cleaning, providing a larger reservoir for mosquito breeding [81]. The average household size in Trapeang Roka village is 4.3, which is significantly higher than those recorded in La Réunion (2.9, [82]) and Italy (2.4, source: Eurostat). A similar pattern was shown for the Indian Ocean island of Mayotte, where more cases occurred in larger households [83] and clustering of cases within households has also been reported for dengue fever [84]. Other local factors such as climate and lifestyle also contributed to this larger estimate. In particular, detailed information on local vector densities could provide a more accurate estimate. Furthermore, the accurate data on the presence of asymptomatic individuals, and those with an undocumented date of symptom onset, provided a unique opportunity to assess their impact on the calculation of 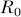. The importance of asymptomatic infections in the spatio-temporal spread of chikungunya has been demonstrated [34] and it has been shown that the movement of asymptomatic individuals alone is sufficient to initiate an epidemic in an immunologically naive population. Therefore, neglecting such individuals when determining the reproduction number may produce unrealistically low estimates, as we have demonstrated using a simple SEIR model. Furthermore, a sensitivity analysis showed that the model is highly influenced by parameter choices, such as the biting rate and mosquito longevity, with estimates of 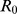 ranging from as low as 3.4 to as high as 7.97. More accurate estimates of such quantities tailored to specific outbreak locations could help to better parameterise mathematical models and reduce calculation errors. In particular, biting rates have been shown to be affected by the disease status of individuals with febrile patients being more attractive to mosquitoes [85]. Such a phenomenon could be further explored using the ABM framework to allow for variable biting rates as a function of disease status throughout the population. Another parameter with great uncertainty is the extrinsic incubation period. The virus has been detected in infected mosquitoes at two days post infection, however, this could be significantly delayed if the mosquito was exposed to a lower viremia. The sensitivity analysis found that small variations in the extrinsic incubation period did not have a large impact on the results, however, a more detailed analysis showed that significantly small 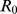 estimates (as low as 2.2) could be obtained for incubation periods of duration 6 days or greater. Better laboratory estimates of such mosquito characteristics could help to identify more realistic incubation times and reduce calculation errors.

Accurate estimates of 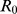 are particularly important in recent times which have seen non-native mosquitoes invade and colonise previously unoccupied regions. The non-native *Aedes albopictus* mosquito was responsible for the first chikungunya outbreak in a temperate region recorded in the Emilia Romagna region of Italy. This outbreak highlighted the need to understand the dynamics of disease introduction into new regions and the impact of biological, human and climatic factors on invasion dynamics. The model presented here provides an estimate for 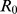 in an immunologically-naive, relatively isolated, population. The large value obtained for 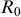 may not be replicated on larger spatial scales, where sustained transmission between towns and cities may be lower than that recorded in a small village with many houses in close proximity. Nevertheless, the model provides insights into the initial dynamics of newly invading pathogens and the importance of closely monitoring new outbreaks for cases that may escape detection. The model indicates the need for accurate monitoring of newly emerging pathogens, or pathogens endemic in tropical areas visited by tourists, in order to identify the route of introduction. This is particularly relevant in the European context, where many chikungunya cases were imported during the Indian Ocean outbreak and many territories can seed European outbreaks with imported cases [86], [87]. The need to develop and implement stochastic models is especially needed in the early stages of such outbreaks, when the number of infected individuals is small. A better understanding of the dynamics of such disease invasions can better inform public health officials and impact control strategies. Models developed in pre-epidemic periods can be used to predict hospital planning needs, the impact of school closure, the financial burden and the cost-effectiveness of mitigation measures.
